# Knowledge and perceptions of donor human milk among university health sciences students in southern Ecuador: a cross-sectional study

**DOI:** 10.3389/fgwh.2026.1796689

**Published:** 2026-05-13

**Authors:** Ana Lizette Rojas-Rodríguez, Josselyne Dayana Imaicela, José Ivo Contreras, Jestin Alejandro Quiroz

**Affiliations:** 1 Department of Health Sciences, Faculty of Health Sciences, Universidad Técnica Particular de Loja (UTPL), Loja, Ecuador; 2 Universidad de Especialidades Espíritu Santo (UEES), Samborondón, Ecuador

**Keywords:** donor human milk, human milk bank, health sciences students, knowledge, perception, Ecuador, breastfeeding, maternal and child health

## Abstract

**Background:**

Donor human milk (DHM) is the safest nutritional alternative when breastfeeding is not possible, particularly for preterm newborns. Health sciences undergraduate students will play a key role in DHM counseling, promotion, and appropriate clinical use; however, evidence on their knowledge and perceptions in Latin America remains limited.

**Objective:**

To assess and compare the knowledge and perceptions regarding donor human milk (DHM) among undergraduate students of Medicine, Nursing, and Nutrition and Dietetics in southern Ecuador, and to examine the association between both variables according to academic program.

**Methods:**

An analytical cross-sectional study was conducted between October 2022 and April 2023 at a university in southern Ecuador. A total of 387 valid questionnaires were analyzed using convenience sampling. Data were collected using three structured instruments: a sociodemographic/academic questionnaire, an *ad hoc* 12-item dichotomous knowledge questionnaire, and an *ad hoc* 15-item dichotomous perception questionnaire. Associations were explored by academic program. The association between knowledge and perception scores was assessed using Spearman's rank correlation coefficient (rs). Adjusted odds ratios (OR) were estimated for knowledge categories by academic program.

**Results:**

Overall, 59% of participants showed low DHM knowledge and 41% high knowledge, with significant differences across academic programs (*p* = 0.007). Perceptions were predominantly favorable (75.2%) and did not differ significantly across sociodemographic or academic groups (*p* > 0.05). A weak but statistically significant positive correlation between knowledge and perception scores was observed in the overall sample (rs = 0.12; *p* = 0.02) and among Nursing (rs = 0.165; *p* = 0.02) and Nutrition and Dietetics students (rs = 0.29; *p* = 0.02), whereas no significant correlation was found among Medicine students (rs = −0.02; *p* = 0.80). Nutrition and Dietetics students showed higher odds of adequate knowledge in categories related to DHM storage (OR = 2.39; 95% CI: 1.36–4.15) and DHM availability, access, and clinical use (OR = 2.03; 95% CI: 1.16–3.55).

**Conclusion:**

Although perceptions toward DHM were favorable, knowledge gaps persist. The association between knowledge and perception was weak and not uniform across programs, highlighting the need to strengthen and standardize undergraduate training to support evidence-based counseling and improve DHM implementation.

## 1 Introduction

Human milk is considered the optimal infant feeding source due to its complex biological composition and its immunological, metabolic, and neurocognitive benefits (1, 2). When breastfeeding is not possible or insufficient, donor human milk (DHM) represents the best therapeutic alternative to ensure optimal nutrition (3, 4), particularly for vulnerable newborns requiring neonatal intensive care. The latest evidence from the Lancet Breastfeeding Series (2023) redefines breastfeeding as a collective societal responsibility, emphasizing that DHM is a critical component of high-quality neonatal care (5).

It is essential to distinguish between DHM and human milk banks (HMBs). DHM refers to milk expressed by a lactating mother for infants who are not her biological children, whereas an HMB is the specialized institution responsible for the safe processing, storage, and distribution of DHM (4). In preterm or low birth weight newborns, DHM significantly reduces the risk of necrotizing enterocolitis (NEC), sepsis, and other severe complications without compromising long-term neurodevelopment (6–9).

The World Health Organization (WHO) and UNICEF (2024) have identified the expansion of human milk bank (HMB) networks as a priority strategy for reducing neonatal mortality (10). However, evidence indicates that the effectiveness and sustainability of these systems may be constrained by knowledge gaps and insufficient training among students and future healthcare professionals (11). Obstetricians and other medical professionals should develop and maintain knowledge and skills to provide anticipatory guidance, perform physical assessments and support the establishment and maintenance of breastfeeding. Nurses, who serve on the front line in maternity and neonatal units, provide direct counselling, collect donor milk, oversee its processing and ensure safe storage. Clinical dietitians are responsible for assessing the infant's nutritional status and fortifying donor milk as needed, together with nurses and physicians they participate in human milk bank advisory committees to monitor operations, provide feedback and guide integration of milk-bank staff into neonatal care (12).

The knowledge, attitude, practice theoretical model proposes that knowledge forms the foundation for developing attitudes and behaviours, while attitudes drive changes in practice. Consistent with this framework, empirical studies of human milk banking among nurses have identified a positive, albeit modest, correlation between knowledge and perception of milk donation, supporting the examination of both dimensions simultaneously (13, 14).

Systematic reviews further demonstrate that human milk donation depends not only on institutional capacity, but also on donor awareness, the quality of professional counseling, and the sociocultural acceptability of the process (15). In this context, strengthening technical and communication competencies during undergraduate training is essential to ensure the appropriate implementation and sustained functioning of HMB networks.

The World Health Organization (WHO) and UNICEF (2024) recommend expanding HMB networks as a key strategy for reducing neonatal mortality (10). However, recent evidence suggests that knowledge gaps and limited training among healthcare students remain primary barriers to strengthening donation systems (11, 15, 16). In Ecuador, while nine milk banks have benefited thousands of newborns, access remains centralized in urban areas (17). Recent national regulations, including the 2025 MSP Manual, emphasize the need for technically prepared professionals to bridge the gap in rural access (18). This study aims to assess knowledge and perceptions regarding DHM among health sciences students in southern Ecuador to inform targeted educational strategies.

Human milk is considered the optimal infant feeding source due to its composition and its immunological, metabolic, and neurocognitive benefits (1). When breastfeeding is not possible or insufficient, donor human milk (DHM) represents the best alternative to ensure optimal nutrition particularly for vulnerable newborns requiring neonatal care (2, 3).

It is important to distinguish between DHM and human milk banks (HMBs). DHM refers to milk expressed by a lactating mother to be consumed by infants who are not her biological children, whereas an HMB is the institution responsible for the safe processing, storage, and distribution of DHM (4). This distinction is relevant because knowledge and perceptions about DHM may influence its acceptability and use, independent of familiarity with how milk banks operate.

In preterm or low birth weight newborns, DHM reduces the risk of necrotizing enterocolitis, sepsis, and other severe complications and may shorten hospital stay (5-7-9). For this reason, the World Health Organization (WHO) recommends expanding HMBs and supporting DHM donation as part of neonatal health strategies and breastfeeding promotion (10). However, evidence from Latin America suggests that knowledge gaps and limited training among healthcare professionals and future providers remain key barriers to strengthening DHM donation systems and ensuring equitable access (11, 15–17).

In Ecuador, DHM is regulated for use in preterm newborns or infants with critical conditions and must come from screened healthy donors. Although nine milk banks have benefited more than 111,000 newborns, access remains limited in vulnerable areas due to their urban distribution (18). In this context, undergraduate training becomes strategic, as students in health sciences programs will play an active role in counseling, clinical indication, and referral processes related to breastfeeding support and DHM use. Medicine, Nursing, and Nutrition and Dietetics were selected because these programs prepare future health professionals with complementary roles in maternal and newborn care, breastfeeding support, nutritional counseling, and the clinical indication or promotion of donor human milk. Assessing these three disciplines jointly may help identify program-specific educational gaps in competencies that are highly relevant for the implementation of human milk banking systems. In addition, previous evidence suggests that knowledge and perception are related but not necessarily equivalent constructs, as favorable attitudes toward donor human milk may coexist with limited technical understanding. Exploring both dimensions together is therefore important to better understand readiness for evidence-based counseling and future professional practice (19–21).

Therefore, the aim of this study was to assess knowledge and perceptions regarding donor human milk (DHM) among undergraduate students of Medicine, Nursing, and Nutrition and Dietetics, and to compare the results by academic program. An analytical cross-sectional design was selected because it allows data to be collected at a single point in time and represents an efficient approach to estimate the prevalence of knowledge gaps and to examine the relationship between knowledge and perceptions in a regional context with limited available evidence.

This is the first analytical cross-sectional study conducted in Ecuador addressing this topic and contributes evidence to the limited literature available in Latin America.

## 2 Materials and methods

### 2.1 Study design and setting

An analytical cross-sectional study was conducted between October 2022 and April 2023 at a university in southern Ecuador. The study aimed to assess knowledge and perceptions regarding donor human milk (DHM) among undergraduate health sciences students and examine differences by academic program and associated factors.

### 2.2 Participants and sampling

The study population consisted of 843 undergraduate students enrolled in the Medicine, Nursing, and Nutrition and Dietetics programs during the study period. The minimum required sample size was calculated using the finite population formula, assuming an expected proportion of 0.5, a 95% confidence level (*Z* = 1.96), a maximum admissible error of 5%, and a design effect of 1, resulting in a minimum of 264 participants. A non-probabilistic convenience sampling strategy was applied.

A total of 387 complete questionnaires were included in the final analysis, corresponding to students from Medicine (*n* = 147; 38.0%), Nursing (*n* = 180; 46.5%), and Nutrition and Dietetics (*n* = 60; 15.5%). Two reminder emails were sent at 15-day intervals to improve the response rate.

Inclusion criteria were: (1) being an undergraduate student with active enrollment in an eligible program, (2) providing electronic informed consent, and (3) completing all questionnaire items. Incomplete forms or responses with inconsistent information were excluded.

### 2.3 Data collection instruments

Data were collected using three structured instruments: (1) a sociodemographic and academic questionnaire, (2) an *ad hoc* knowledge questionnaire on donor human milk (DHM), and (3) an *ad hoc* perception questionnaire on DHM.

Given the limited availability of validated instruments specifically designed to simultaneously assess knowledge and perceptions regarding DHM among undergraduate health sciences students in the Latin American context, the research team developed both the knowledge and perception questionnaires for this study. Item development was informed by a review of the scientific literature and international technical guidance on DHM donation and human milk bank procedures, including recommendations from the Latin American and Caribbean Human Milk Bank Network (REDLACMA).

#### 2.3.1 Sociodemographic and academic questionnaire

The sociodemographic and academic questionnaire collected information on sex, place of residence, marital status, parental status (children), academic program, and academic level according to the institutional curriculum.

#### 2.3.2 Knowledge questionnaire on donor human milk (DHM)

Knowledge regarding DHM was assessed using an *ad hoc* 12-item questionnaire with dichotomous responses (Yes/No), specifically developed for this exploratory study. The items addressed key technical and procedural aspects of DHM and human milk banks, including knowledge of DHM availability, donor eligibility and screening criteria, safety procedures (e.g., pasteurization), storage and handling conditions, and clinical indications for DHM use, particularly in preterm newborns or when breastfeeding is not possible.

Example questions included: “What is the standard method for human milk disinfection?” and “What is the maximum storage time under frozen conditions?”.

#### 2.3.3 Perception questionnaire on donor human milk (DHM)

Perceptions regarding DHM were assessed using an *ad hoc* 15-item questionnaire with dichotomous responses (Yes/No), designed specifically for this exploratory study. The instrument explored complementary perception domains, including beliefs about the nutritional value and biological quality of DHM (e.g., perceived loss of nutrients or calories during storage and preservation of protective components), perceived clinical benefits (especially for preterm or ill newborns), and the social, emotional, and cultural acceptability of donation (e.g., willingness to donate, preference to receive milk only from one's own mother, perceived influence of religion, and potential barriers related to milk expression or maternal health).

Example statements included: “I consider donor human milk biologically superior to infant formula” and “I would be willing to recommend milk donation to a lactating mother.” The full list of items is provided as Supplementary Material S1.

### 2.4 Content validity and pilot testing

Content validity was assessed by a panel of five experts in breastfeeding, maternal and child health, nutrition, and public health. Experts were selected through purposive sampling based on predefined eligibility criteria: (1) at least five years of professional or academic experience in maternal–child health or breastfeeding-related fields, (2) participation in teaching, clinical practice, or research activities related to human milk, breastfeeding, or neonatal nutrition, and (3) previous involvement in instrument development, guideline implementation, or evaluation of health programs. The selection aimed to ensure disciplinary diversity and adequate domain expertise rather than statistical representativeness. A formal expert scoring system (e.g., the Fehring model) was not applied; eligibility criteria were established *a priori* to ensure methodological rigor and domain expertise.

A structured Microsoft Excel matrix was developed including: (a) study objectives, (b) operational definitions of the assessed variables, (c) each questionnaire item, (d) thematic domain assignment (knowledge or perception), and (e) evaluation criteria. The matrix was distributed individually to each expert along with standardized instructions to ensure independent judgment.

Experts independently evaluated item relevance, clarity, and conceptual adequacy using a 4-point ordinal scale (1 = not relevant, 4 = highly relevant), providing qualitative feedback when necessary. The research team systematized the evaluations and calculated the Content Validity Index (CVI). The global CVI was 0.83 for the knowledge questionnaire and 0.85 for the perception questionnaire, supporting their adequacy for exploratory and descriptive purposes.

A pilot test was subsequently conducted with 20 students from the same institution and with characteristics comparable to the target population to assess item clarity and comprehension. The pilot was used exclusively to verify semantic understanding and contextual adequacy; no psychometric analyses were performed, and pilot data were not included in the final analyses. No major modifications were required.

Internal consistency (Cronbach's alpha) was not estimated because both instruments were developed as *ad hoc* questionnaires for exploratory and descriptive purposes and covered conceptually heterogeneous domains. Items were not intended to form a unidimensional psychometric scale measuring a single latent construct; therefore, Cronbach's alpha could be uninformative or potentially misleading. Accordingly, content validity evidence was prioritized as the main methodological quality criterion supporting item adequacy.

### 2.5 Data collection procedure

Questionnaires were administered online using the institutional platform, ensuring participant anonymity. The survey invitation, study information sheet, informed consent form, and access link were distributed via official institutional email to eligible students. Distribution was supported by the Federation of University Student Centers, which facilitated dissemination of the invitation but had no role in data collection, access to responses, or data analysis, in order to reduce potential coercion and preserve voluntary participation**.**

Electronic informed consent was mandatory prior to accessing the questionnaires. To minimize duplicate responses, institutional authentication was required, allowing only one submission per participant. Data were stored in anonymized form in a secure, password-protected institutional repository accessible only to the research team.

### 2.6 Variables and operational definitions

#### 2.6.1 Knowledge about donor human milk

Knowledge was evaluated objectively based on concordance with international technical recommendations regarding DHM donation and use. Each item was scored as 1 (correct) or 0 (incorrect), resulting in a total score ranging from 0 to 12, where higher scores indicated greater knowledge. A normative threshold of 70% correct responses was applied to classify knowledge as high (≥9 correct answers) or low (<9 correct answers).

#### 2.6.2 Perceptions about donor human milk

Perception was considered a subjective and attitudinal construct. The perception questionnaire consisted of 15 dichotomous items, which were summed to obtain a total score ranging from 0 to 15, with higher scores reflecting more favorable perceptions. Since universal normative cutoffs are not available, perception was classified using a sample-based approach, applying the median score as the cutoff to define favorable (≥ median) vs. unfavorable (< median) perception.

#### 2.6.3 Construction of thematic categories (category-based analysis)

Because both questionnaires included conceptually heterogeneous items, an *a priori* thematic grouping was performed to support category-specific analyses. This grouping was conducted through expert judgment by a faculty member from the Nutrition and Dietetics program with expertise in breastfeeding and DHM, who did not participate in data collection or statistical analysis and contributed only to conceptual instrument review.

Knowledge categories included: (1) donor human milk storage and handling, (2) clinical benefits for preterm infants, (3) donation eligibility criteria, and (4) availability, access, and clinical use. Perception categories included: (1) perceived nutritional value of DHM, (2) perceived clinical benefits of DHM, (3) social, emotional, and cultural acceptability of donating/receiving DHM, and (4) perceptions regarding appropriate recipients of DHM.

For each category, item scores were summed to generate a category-level score, and participants were classified into adequate vs. inadequate knowledge (or favorable vs. unfavorable perception) according to the predefined scoring criteria.

Adjusted odds ratios (OR) and 95% confidence intervals (95% CI) were estimated using multivariable logistic regression models to evaluate the association between academic program and category-level outcomes, adjusting for sex, religion, and academic level**.**

### 2.7 Ethical considerations

The study was approved by the Human Research Ethics Committee of Universidad Técnica Particular de Loja (UTPL-CEISH-2022-PG11). Participation was voluntary, and confidentiality and anonymity were ensured. No personally identifiable information was collected.

### 2.8 Statistical analysis

A descriptive analysis was conducted using absolute and relative frequencies. Associations between sociodemographic and academic characteristics and knowledge or perception levels were assessed using the Chi-square test (*χ*²). The magnitude of association was estimated using odds ratios (ORs) with 95% confidence intervals (95% CIs). Adjusted odds ratios (ORs) and 95% confidence intervals (95% CIs) were estimated using multivariable logistic regression models to evaluate the association between academic program and adequate knowledge/perception outcomes across predefined thematic categories. Medicine was used as the reference category for all regression analyses, and models were adjusted for sex, religion, and academic level.

The association between knowledge and perception scores was evaluated using Spearman's rank correlation coefficient (rs), given the ordinal nature of the variables and the absence of normality assumptions. Correlations were calculated by academic program and in the overall sample, and the strength and direction of the association were interpreted based on the rs value.

Statistical significance was set at *p* < 0.05. All analyses were conducted using IBM SPSS Statistics® version 25.

## 4 Results

### 4.1 The final sample consisted of 387 health sciences students

Regarding sociodemographic characteristics, a marked female predominance was observed (80.6%) compared to males (19.4%). The entire study population (100%) reported residing in urban areas.

Concerning marital status and family structure, the vast majority of participants reported not having a stable partner (92.8%) and having no children (94.6%). In the academic field, the most represented group was Nursing students (46.5%), followed by Medicine (38.0%) and Nutrition and Dietetics (15.5%). Finally, regarding academic progress, more than two-thirds of the sample (67.7%) were in their early years (1st to 5th semester), while the remaining 32.3% belonged to advanced levels. Table 1.

**Table 1:** Sociodemographic and academic characteristics of participants.

Characteristics	*N*	%
Sex		
Male	75	19.4
Female	312	80.6
Place of residence		
Urban	387	100.0
Marital status		
Without partner (single/divorced)	359	92.8
With partner (married/cohabiting)	28	7.2
Children		
Yes	21	5.4
No	366	94.6
Academic program		
Medicine	147	38.0
Nursing	180	46.5
Nutrition and Dietetics	60	15.5
Academic level		
Early years (1st–5th semester)	262	67.7
Advanced years (6th–10th semester)	125	32.3

a *n: absolute* frequency; % relative frequency. Academic level: Early Years (1st–5th semesters, representing the pre-clinical or basic science stage) and Advanced Years (6th–10th semesters, representing the clinical training stage). Data were collected through self-administered instruments.

### 4.2 Association between knowledge of donor human milk and sociodemographic and academic characteristics

A total of 387 valid questionnaires were analyzed. Most participants were female (80.6%) and single (92.8%). Overall, 59% of students showed a low level of knowledge about donor human milk (DHM), while 41% showed a high level of knowledge. Significant differences were observed according to academic program (*p* = 0.007), with the highest proportion of low knowledge in Medicine (66.7%), followed by Nursing (58.3%) and Nutrition and Dietetics (43.3%).

Differences in knowledge level according to religion were identified using the chi-square test (*p* = 0.037). A higher proportion of high knowledge was observed among students reporting other religions (52.2%) compared with those reporting Catholic affiliation (38.4%). This association should be interpreted cautiously, as the “other religions” category grouped heterogeneous denominations and represented a smaller subgroup, which may affect estimate stability.

Knowledge level also differed according to academic level (*p* = 0.008), whereas no statistically significant differences were observed by sex (*p* = 0.718) (Table 2).

**Table 2:** Sociodemographic characteristics associated with knowledge of donor human milk.

Characteristics	Low (59%) *n* %		High (41%) *n* %		*p*-value
Sex					
Male	43	57.3	32	42.7	0.718
Female	186	59.6	126	40.4	
Religion					
Catholic	197	61.6	123	38.4	0.037
Other	32	47.7	34	52.2	
Academic level (semester)					
Early years (1st–5th semester)	167	63.7	95	36.3	0.008
Advanced years (6th–10th semester)	62	49.6	63	50.4	
Academic program					
Medicine	98	66.7	49	33.3	0.007
Nursing	105	58.3	75	41.7	
Nutrition and Dietetics	26	43.3	34	56.7	

Data are presented as *n* (%). *p*-values were obtained using chi-square tests. Low and high knowledge were classified using a 70% cutoff, with high knowledge defined as ≥9 correct responses out of 12 items.

### 4.3 Association between perceptions of donor human milk and sociodemographic and academic characteristics

Overall, perceptions toward donor human milk were predominantly favorable in the study population (75.2%). No statistically significant differences in perception level (favorable vs. unfavorable) were observed according to sex (*p* = 0.283), religion (*p* = 0.151), academic level (*p* = 0.313), or academic program (*p* = 0.188) (Table 3).

**Table 3:** Association between perception of donor human milk (DHM) and sociodemographic and academic characteristics (chi-square analysis).

Characteristics	Unfavorable *n* (%)	Favorable *n* (%)	*p*-value
Sex			
Male	15 (20.0)	60 (80.0)	0.283
Female	81 (26.0)	231 (74.0)	
Religion			
Catholic	84 (26.3)	236 (73.7)	0.151
Other	12 (17.9)	55 (82.1)	
Academic level			
Initial	69 (26.3)	193 (73.7)	0.313
Advanced	27 (21.6)	98 (78.4)	
Program			
Medicine	29 (19.7)	118 (80.3)	0.188
Nursing	51 (28.3)	129 (71.7)	
Nutrition and Dietetics	16 (26.7)	44 (73.3)	

Percentages are calculated over the total sample. Statistical significance was set at *p* < 0.05.

### 4.4 Association between knowledge and perceptions of donor human milk

To examine the association between donor human milk (DHM) knowledge and perception scores, Spearman's rank correlation coefficient (rs) was calculated by academic program and in the overall sample. A weak but statistically significant positive association was observed among Nursing students (rs = 0.165; *p* = 0.02), Nutrition and Dietetics students (rs = 0.29; *p* = 0.02), and in the overall sample (rs = 0.12; *p* = 0.02), indicating that higher knowledge scores were modestly associated with more favorable perceptions. In contrast, no statistically significant association was found among Medicine students (rs = −0.02; *p* = 0.80) (Table 4).

**Table 4:** Association between knowledge and perception regarding donor human milk (Spearman correlation) according to academic program.

Statistic	Nursing (*n* = 180)	Medicine (*n* = 147)	Nutrition and Dietetics (*n* = 60)	Total (*n* = 387)
Spearman's rs	0.165	−0.02	0.29	0.12
P	0.02^*^	0.80	0.02^*^	0.02^*^

Statistical significance was set at *p* < 0.05. The symbol (*) indicates statistically significant values (*p* < 0.05).

### 4.5 Association between academic program and adequate knowledge across categories

As shown in Table 5, adjusted odds ratio analyses revealed differences in adequate knowledge across academic programs in two of the four evaluated categories.

**Table 5:** Adjusted odds ratios for adequate knowledge across knowledge categories, by academic program.

Knowledge categories	Nursing OR(CI95%)	Medicine OR(CI 95%)	Nutrition and Dietetics OR (CI 95%)	*p*-value
Donor human milk storage	0.81 (0.53–1.22)	0.75 (0.49–1.15)	2.39 (1.36–4.15)	0.008^*^
Clinical benefits for preterm infants	0.94 (0.61–1.43)	0.87 (0.56–1.34)	0.96 (0.77–1.21)	0.47
Donation requirements and eligibility criteria	1.42 (0.94–2.16)	0.66 (0.42–1.01)	1.06 (0.66–1.88)	0.15
Availability, access, and clinical use	1.18 (0.77–1.79)	0.54 (0.34–0.85)	2.03 (1.16–3.55)	0.006^*^

OR, odds ratio; CI, confidence interval. Statistical significance was set at *p* < 0.05. The symbol (*) indicates statistically significant values (*p* < 0.05).

In the donor human milk storage category, students from Nutrition and Dietetics showed higher odds of adequate knowledge (OR = 2.39; 95% CI: 1.36–4.15; *p* = 0.008), while Nursing (OR = 0.81; 95% CI: 0.53–1.22; *p* > 0.05) and Medicine (OR = 0.75; 95% CI: 0.49–1.15; *p* > 0.05) showed no statistically significant differences.

No statistically significant associations were observed between academic programs in the clinical benefits for preterm infants' category (*p* = 0.47) or in the donation requirements and eligibility criteria category (*p* = 0.15).

In the availability, access, and clinical use category, Medicine showed lower odds of adequate knowledge (OR = 0.54; 95% CI: 0.34–0.85; *p* = 0.006), whereas Nutrition and Dietetics showed higher odds (OR = 2.03; 95% CI: 1.16–3.55; *p* = 0.006). Nursing showed no statistically significant association (OR = 1.18; 95% CI: 0.77–1.79; *p* > 0.05).

### 4.6 Perception of donor human milk by academic program

As shown in Table 6, adjusted odds ratio (OR) analyses did not identify statistically significant differences in perception categories regarding donor human milk (DHM) across academic programs, as all 95% confidence intervals (95% CI) included the null value and all *p*-values were > 0.05.

**Table 6:** Adjusted odds ratios by academic program according to perception categories regarding dono human milk (DHM).

Categorie	Nursing OR (CI 95%)	Medicine OR (CI 95%)	Nutrition and Dietetics OR (CI 95%)	*p*-value
Perception of nutritional value	1.08 (0.72–1.62)	0.95 (0.64–1.47)	0.97 (0.64–1.47)	0.89
Perception of the clinical benefits of donation	0.93 (0.60–1.44)	1.05 (0.67–1.65)	0.72 (0.47–1.07)	0.95
Social, emotional, and cultural acceptance of donating or receiving human milk	0.71 (0.47–1,07)	1.43 (0.95–2.17)	0.98 (0.56–1.71)	0.20
Percep ion regarding who should receive donated human milk	1.69 (0.84–3.38)	0.51 (0.23–1.12)	1.10 (0.43–2.79)	0.22

OR odds ratio; CI, confidence interval Statistically significant *p* < 0.05.

For the category *perceived nutritional value of DHM*, no significant associations were observed for Nursing (OR = 1.08; 95% CI: 0.72–1.62), Medicine (OR = 0.95; 95% CI: 0.64–1.47), or Nutrition and Dietetics (OR = 0.97; 95% CI: 0.64–1.47) (*p* = 0.89).

Similarly, the category *perceived clinical benefits of donation* showed no statistically significant differences across programs, including Nursing (OR = 0.93; 95% CI: 0.60–1.44), Medicine (OR = 1.05; 95% CI: 0.67–1.65), and Nutrition and Dietetics (OR = 0.72; 95% CI: 0.47–1.07) (*p* = 0.95).

Regarding *social, emotional, and cultural acceptance of donating or receiving human milk*, none of the programs showed significant associations (Nursing OR = 0.71; 95% CI: 0.47–1.07; Medicine OR = 1.43; 95% CI: 0.95–2.17; Nutrition and Dietetics OR = 0.98; 95% CI: 0.56–1.71) (*p* = 0.20).

Finally, for the category *perception regarding appropriate recipients of DHM*, no statistically significant differences were identified across programs (Nursing OR = 1.69; 95% CI: 0.84–3.38; Medicine OR = 0.51; 95% CI: 0.23–1.12; Nutrition and Dietetics OR = 1.10; 95% CI: 0.43–2.79) (*p* = 0.22).

## 5 Discussion

The present study provides novel evidence in Latin America by examining the knowledge and perceptions of undergraduate health sciences students, a group that has been less explored compared to the extensive literature focused on mothers and clinical professionals.

In general terms, the findings show that technical knowledge about DHM was low across the three academic programs evaluated, particularly in Medicine, followed by Nursing, while Nutrition and Dietetics showed the best performance. In contrast, perceptions toward donation were mostly favorable and homogeneous across academic and sociodemographic groups. This dissociation between positive perceptions and limited technical knowledge coincides with other contemporary studies where, even when favorable attitudes exist, cognitive or behavioral barriers persist, such as a preference for known recipients or reluctance to use milk from another mother (22–24). Ensuring that this positive attitude is backed by technical mastery is crucial for future clinical practice.

Additionally, the absence of statistically significant differences in perception categories across academic programs suggests that attitudes toward donor human milk are not primarily determined by formal academic training. Despite variability in technical knowledge, students showed similar levels of acceptance regarding nutritional value, clinical benefits, and social acceptability of donation. This pattern has been previously described, where favorable attitudes coexist with limited or heterogeneous knowledge, indicating that cognitive and attitudinal components develop through partially independent pathways (19, 20, 25, 26). In this context, sociocultural factors, personal beliefs, and perceived safety may influence acceptance more strongly than technical information acquired during training (22, 26).

Spearman correlation analysis allowed further exploration of this relationship and identified differences according to academic program. A positive association between knowledge and perception was observed among Nursing and Nutrition and Dietetics students, as well as in the overall sample, suggesting that greater conceptual mastery may be accompanied by more favorable attitudes toward donation. However, this relationship was not observed among Medicine students, indicating that in this group, favorable perceptions may be influenced by factors other than formal knowledge, such as early clinical experiences, partial information, or personal beliefs (19, 20). Addressing this gap is essential, as clinical counseling that is insufficiently grounded in technical knowledge may contribute to the unnecessary use of commercial formulas, which are frequently actively promoted to healthcare professionals (6). Therefore, strengthening structured teaching on human milk banking and the clinical use of DHM in undergraduate training is essential across all programs.

The analysis by specific knowledge categories provided additional relevant information. In the categories related to DHM conservation and to availability, access, and clinical use, Nutrition and Dietetics students showed a higher probability of presenting adequate knowledge. This finding is consistent with the educational focus of this program, which is more oriented toward handling, conservation, and food safety processes. In contrast, Medicine and Nursing showed limitations in these categories, which is particularly relevant given their clinical role in indicating and ensuring the safe use of DHM. Current international organizations recommend that future professionals receive practical training in collection, storage, transportation, and pasteurization, integrating biosafety and quality criteria (2). Standardizing this information across health sciences programs is necessary to ensure consistent evidence-based advice according to global implementation frameworks (4).

One of the most critical knowledge gaps pertains to the therapeutic efficacy of DHM in the neonatal intensive care unit (NICU). While students value DHM, many do not fully recognize its decisive role in reducing severe morbidity. Current high-quality evidence, particularly the 2024 Cochrane systematic review update, demonstrates that feeding very preterm infants with DHM instead of formula reduces the risk of necrotizing enterocolitis (NEC) by approximately 50% (3). Furthermore, recent studies highlight that DHM supports intestinal maturation and reduces late-onset sepsis, reinforcing its role as a biological medicine rather than just a nutritional source (7). Without a deep understanding of these benefits, future physicians may fail to prioritize DHM over commercial formula, missing the opportunity to use “personalized medicine” through human milk. Education must emphasize these clinical outcomes to ensure DHM is prescribed appropriately (7).

The variability found across programs reinforces the need to standardize curricular content on donation and handling of human milk, so that future professionals develop homogeneous and evidence-based competencies. This is especially relevant considering that acceptance and practice of donation are conditioned by the level of knowledge, cultural beliefs, and trust in the healthcare system (19, 23).

Receiving clear and adequate information significantly increases willingness to donate, which directly impacts the availability of DHM for vulnerable newborns (22, 24, 27).

As emphasized in the 2023 Lancet Breastfeeding Series, breastfeeding and the use of donor human milk should be framed as a collective societal responsibility rather than solely an individual maternal choice (5).In this context, strengthening technical competence during undergraduate training represents a strategic investment in a healthcare system capable of promoting equitable access to DHM, counteracting formula marketing pressures, and advancing progress toward the 2030 global nutrition targets through evidence-based neonatal care.

Finally, it should be considered that DHM constitutes an essential component of the right to safe and equitable nutrition, which requires competent professionals for its promotion and management (28, 29).

### 5.1 Implications for maternal and child health

The findings of the present study have relevant implications for strengthening maternal and child health and breastfeeding support strategies. Although attitudes toward DHM were favorable, the gaps identified in technical knowledge suggest that acceptance alone does not guarantee safe, timely, and evidence-based use. Therefore, it is crucial to consolidate structured university training on DHM and human milk banks, incorporating technical content (donation criteria, biosafety, conservation processes, and clinical use) as well as ethical and sociocultural components that influence decision-making.

Likewise, the role of health professionals as a source of communication and information exchange for potential donors has been previously reported (23), which reinforces the need to strengthen in students competencies in counseling, education, and the promotion of donation as part of a comprehensive approach to neonatal health. In this sense, integrating these contents from the undergraduate level may foster an informed, safe, and sustainable donation culture, contributing to improved DHM availability for vulnerable newborns.

This pioneering study in Ecuador and the Andean region highlights the importance of promoting educational interventions aimed at reducing training gaps and strengthening the potential role of future health professionals in the promotion and appropriate use of donor human milk.

## 6 Conclusions

This study examined the levels of knowledge and perceptions regarding donor human milk (DHM) among undergraduate health sciences students and evaluated the relationship between both variables according to academic program. A meaningful dissociation was identified between the cognitive and attitudinal components. While perceptions toward donation were predominantly favorable and consistent across disciplines, technical knowledge was limited and varied significantly according to disciplinary training.

Importantly, the relationship between knowledge and perception was not uniform. A positive association was observed among Nursing and Nutrition and Dietetics students, suggesting that stronger conceptual mastery may reinforce favorable attitudes. In contrast, among Medicine students, positive perceptions appeared to be independent of technical knowledge.

The presence of a pro-donation attitude among future health professionals represents a valuable foundation; however, it is insufficient on its own to ensure safe, evidence-based clinical practice. The knowledge gaps identified in critical areas particularly storage procedures, disinfection processes, and donor eligibility criteria highlight the need to strengthen and standardize undergraduate education on human milk banking and DHM management.

## 7 Limitations

This study has several limitations. First, the cross-sectional design precludes establishing causal relationships between knowledge and perception. Second, the use of non-probabilistic convenience sampling in a single university may have introduced selection bias and limits the generalizability of the findings to other institutions or regions with different academic, cultural, or educational characteristics. Additionally, students with greater interest in breastfeeding or maternal child -health topics may have been more likely to participate, potentially influencing the observed levels of knowledge and perception.

Although the questionnaires demonstrated adequate content validity, they were *ad hoc* instruments without full psychometric validation, which may affect measurement precision. The use of self-administered questionnaires may also have introduced information bias, particularly social desirability bias, as participants might have reported more favorable attitudes toward donor human milk than their actual beliefs.

Finally, unequal sample sizes across academic programs, particularly in Nutrition and Dietetics, may have reduced the stability of certain estimates and contributed to wider confidence intervals in some analyses. Therefore, results should be interpreted with appropriate caution.

Despite these limitations, the findings provide a relevant indicator of existing educational gaps among undergraduate health sciences students in Ecuador.

## Data Availability

The data will be made available upon reasonable request.
