# Supplementary material for: Knowledge and perceptions of donor human milk among university health sciences students in southern Ecuador: a cross-sectional study
Source: Front Glob Womens Health. 2026 May 13;7:1796689. doi: 10.3389/fgwh.2026.1796689 (PMC13212352; doi:10.3389/fgwh.2026.1796689)
Supplement: Supplementary file 1 [file Datasheet1.pdf]

SUPPLEMENTARY MATERIAL

DATA COLLECTION INSTRUMENTS

SECTION 1. SOCIODEMOGRAPHIC AND ACADEMIC QUESTIONNAIRE

Dear participant: Please read each question carefully and answer according to your sociodemographic and academic characteristics. Your responses are confidential and will be used exclusively for research purposes. Please answer as honestly as possible. Thank you for your participation.

1. Place of residence

☐ Urban

☐ Rural

2. Marital status

☐ Single

☐ Married or living with a partner

3. Do you have children?

☐ Yes

☐ No

If yes, how many? _______

4. Religion

☐ Catholic

☐ Evangelical

☐ Adventist

☐ None

☐ Other: __________

5. Which academic program are you currently enrolled in?

☐ Medicine

☐ Nursing

☐ Nutrition and Dietetics

6. Please write your current academic level/semester (number): _______

SECTION 2. PERCEPTION QUESTIONNAIRE ON DONOR HUMAN MILK

Dear participant: Please read each statement carefully and choose the answer that best reflects your opinion. There are no right or wrong answers. Your responses are confidential and will be used exclusively for research purposes. Thank you for your participation.

1. Do you believe that donor human milk contains all essential nutrients needed for a healthy life and complete infant development?

☐ Yes

☐ No

2. Do you believe that donor human milk loses calories when stored?

☐ Yes

☐ No

3. Do you believe that donating human milk causes the loss of protective components that support neurodevelopment?

☐ Yes

☐ No

4. Do you believe that donor human milk provides the same benefits to preterm newborns who receive it?

☐ Yes

☐ No

5. Do you believe that human milk banks are a viable alternative for women who cannot breastfeed?

☐ Yes

☐ No

6. Do you believe that donating human milk can save babies' lives?

☐ Yes

☐ No

7. Do you believe that donating human milk is also beneficial for maternal health because it reduces breast engorgement?

☐ Yes

☐ No

8. Do you believe that donating human milk affects the nutritional status of the donor mother?

☐ Yes

☐ No

9. Do you believe that in cases of human milk overproduction it is advisable to donate?

☐ Yes

☐ No

10. Do you believe that babies should only receive their own mother's milk and not milk donated by other mothers?

☐ Yes

☐ No

11. Do you believe that mechanical expression (using manual breast pumps) may cause fear in mothers?

☐ Yes

☐ No

12. Do you believe that religion influences human milk donation?

☐ Yes

☐ No

13. If you were to donate, would you feel happy knowing that your donated milk could be the reason for the survival of a sick infant?

☐ Yes

☐ No

14. Do you believe that milk should only be donated to family members?

☐ Yes

☐ No

15. Do you believe that milk should only be donated to family friends?

☐ Yes

☐ No

SECTION 3. KNOWLEDGE QUESTIONS ON DONOR HUMAN MILK

Dear participant: Please read each question carefully and answer based on your knowledge. Your responses are confidential and will be used exclusively for research purposes. Thank you for your participation.

1. Have you previously received information about human milk banks?

☐ Yes

☐ No

2. Do you know that feeding preterm infants with donor human milk has more benefits than feeding them with formula?

☐ Yes

☐ No

3. Do you know that when a mother cannot breastfeed or milk supply is insufficient due to illness, donor human milk can be used as an alternative?

☐ Yes

☐ No

4. Do you know that women who donate human milk need to undergo a medical evaluation?

☐ Yes

☐ No

5. Do you know that after feeding her baby, a mother who still has milk can donate the remaining surplus?

☐ Yes

☐ No

6. For preterm newborns, do you know that feeding with donor human milk can reduce the incidence of heart diseases?

☐ Yes

☐ No

7. Do you know that donor human milk is stored in a human milk bank?

☐ Yes

☐ No

8. Do you know that donor human milk can be given to an infant without the need for disinfection?

☐ Yes

☐ No

9. Do you know that human milk donation has been widely used to feed preterm infants in hospitals?

☐ Yes

☐ No

10. Do you know that there are human milk banks in our country to collect and store donor human milk?

☐ Yes

☐ No

11. Do you know that human milk donors need regular medical tests?

☐ Yes

☐ No

12. Do you know that human milk must be transported under refrigeration?

☐ Yes

☐ No
